# Nontarget organism effects tests on eCry3.1Ab and their application to the ecological risk assessment for cultivation of Event 5307 maize

**DOI:** 10.1007/s11248-013-9778-4

**Published:** 2014-01-10

**Authors:** Andrea Burns, Alan Raybould

**Affiliations:** 1 Syngenta Crop Protection, LLC, 3054 East Cornwallis Road, Post Office Box 12257, Research Triangle Park, NC 27709 USA; 2 Jealott’s Hill International Research Centre, Syngenta, Bracknell, Berkshire, RG22 4JN UK

**Keywords:** Ecological risk assessment, Hypothesis testing, Hazard quotient, Bt crops

## Abstract

Event 5307 transgenic maize produces the novel insecticidal protein eCry3.1Ab, which is active against certain coleopteran pests such as Western corn rootworm (*Diabrotica virgifera virgifera*). Laboratory tests with representative nontarget organisms (NTOs) were conducted to test the hypothesis of no adverse ecological effects of cultivating Event 5307 maize. Estimates of environmental eCry3.1Ab concentrations for each NTO were calculated from the concentrations of eCry3.1Ab produced by 5307 maize in relevant plant tissues. Nontarget organisms were exposed to diets containing eCry3.1Ab or diets comprising Event 5307 maize tissue and evaluated for effects compared to control groups. No statistically significant differences in survival were observed between the control group and the group exposed to eCry3.1Ab in any organism tested. Measured eCry3.1Ab concentrations in the laboratory studies were equal to or greater than the most conservative estimates of environmental exposure. The laboratory studies corroborate the hypothesis of negligible ecological risk from the cultivation of 5307 maize.

## Introduction

Syngenta’s Event 5307 transgenic maize (Agrisure Duracade™ corn) produces the novel insecticidal protein eCry3.1Ab, an engineered chimera of Cry1Ab and modified Cry3A, which are both derived from *Bacillus thuringiensis*. In screening assays conducted with leaf tissue of 5307 maize, or with microbially produced eCry3.1Ab, no activity was observed against any Lepidopteran tested (*Agrotis ipsilon*—black cutworm; *Helicoverpa zea*—corn earworm; *Spodoptera frugiperda*—fall armyworm; *Heliothis virescens*—tobacco budworm; *Ostrinia nubilalis*—European corn borer; and *Pectinophora gossypiella*—pink bollworm). Activity was observed only against certain coleopteran pests in the Chrysomelidae, such as the Western corn rootworm (*Diabrotica virgifera* *virgifera*) (Vlachos and Huber 2011).

Syngenta’s Event MIR604 transgenic maize, which produces a modified Cry3A (mCry3A), also has resistance to Western corn rootworm. Walters et al. (2010) generated data corroborating the hypothesis that eCry3.1Ab can interact with different binding sites than the activated form of mCry3A in the gut brush border membrane of the Western corn rootworm. Therefore, mCry3A and eCry3.1Ab may have different modes of action, which is an advantageous strategy for insect resistance management (Bravo and Soberón 2008).

Prior to approving transgenic crops for commercial cultivation, regulatory agencies assess the environmental risk of cultivating the genetically modified (GM) crop. Laboratory studies are conducted to expose representative nontarget organisms (NTOs) directly to the GM plant material or to concentrations of the insecticidal protein that exceed the concentrations to which they will be potentially exposed in the field. These laboratory studies are conducted to test the hypothesis of no adverse ecological effects from cultivation of the crop. Following a tiered testing approach, if direct exposure to elevated concentrations of the insecticidal protein indicates no adverse effects, then no further testing is required to conclude negligible ecological risk from exposure to protein concentrations resulting from cultivation of the crop. If adverse effects are observed, additional laboratory testing where organisms are exposed to more realistic concentrations of the purified Bt protein or plant material may be conducted if further characterisation of risk is deemed necessary for decision-making. Meta-analysis has indicated that laboratory studies conducted to assess the effects of Bt crops are predictive of, if not more conservative than, field level effects (Duan et al. 2010).

This paper presents estimates of environmental concentrations of eCry3.1Ab via the cultivation of Event 5307 maize and of no-observed-adverse-effect-concentrations from NTO laboratory studies. This information is combined to assess the ecological risk of cultivating Event 5307 maize.

## Materials and methods

### eCry3.1Ab test substances

During the development of Event 5307 maize several NTO effects tests were conducted with plant or microbially produced test substances (Tables 1, 2) containing eCry3.1Ab. To achieve higher eCry3.1Ab test concentrations than are possible using plant-derived test substances, test substance was produced by expressing the *ecry3.1Ab* gene in *Escherichia coli* and purifying the resulting eCry3.1Ab. An evaluation of equivalence (Raybould et al. 2013) comprising comparisons of apparent molecular weight, antibody cross-reactivity, bioactivity against a sensitive insect species, and glycosylation status concluded that effects tests using the microbial produced test substance will conservatively predict the effects of eCry3.1Ab in Event 5307 maize (Vlachos and Huber 2011).

**Table 1:** Species used in effects tests of eCry3.1Ab

Test species	Common name	Order: family	Life stage	Group represented
*Coleomegilla maculata*	Pink-spotted ladybird beetle	Coleoptera: Coccinellidae	Larva	Foliar non-target arthropods
*Orius laevigatus*	Flower bug	Hemiptera: Anthocoridae	Nymph	Foliar non-target arthropods
*Aleochara bilineata*	Rove beetle	Coleoptera: Staphylinidae	Adult	Soil-dwelling invertebrates
*Poecilus cupreus*	Carabid beetle	Coleoptera: Carabidae	Larva	Soil-dwelling invertebrates
*Eisenia fetida*	Earthworm	Haplotaxida: Lumbricidae	Adult	Soil-dwelling invertebrates
*Apis mellifera*	Honey bee	Hymenoptera: Apidae	Brood	Pollinators
*Colinus virginianus*	Bobwhite quail	Galliformes: Phasianidae	Juvenile	Wild birds
*Mus musculus*	Mouse	Rodentia: Muridae	Young adult	Wild mammals
*Gammarus fasciatus*	Freshwater shrimp	Amphipoda: Gammaridae	Adult	Freshwater invertebrates
*Ictalurus punctatus*	Channel catfish	Siluriformes: Ictaluridae	Juvenile	Fish (farmed and wild)

**Table 2:** Summary of designs of effects tests for eCry3.1Ab

Test organism	Source of eCry3.1Ab	Exposure route	Intended bioassay conditions	Duration (days)	Sample size^a^	Guideline or protocol
*C. maculata*	Microbial	Artificial diet	23–27 °C; 40–80 % RH; 16 h L/8 h D	21	40 × 1	OPPTS 885.4340 (1996c)
*O. laevigatus*	Microbial	Artificial diet	24.5–26.5 °C; 65–74 % RH; 16 h L/8 h D	12	40 × 1	OPPTS 885.4340 (1996c)
*A. bilineata*	Microbial	Artificial diet	18–22 °C; 60–90 % RH; 16 h L/8 h D	79^b^	4 × (10♂ + 10♀)	Grimm et al. (2000)
*P. cupreus*	Microbial	Artificial diet	19–21 °C; 60–91 % RH; 16 h L/8 h D	57^b^	40 × 1	Heimbach (1992)
*E. fetida*	Microbial	Artificial soil	20–22 °C; 49–55 % RH; 24 h L	14	4 × 10	OECD 207 (1984)
*A. mellifera*	Microbial	Sucrose solution	Field conditions	24^b^	4 × (100 eggs + 100 larvae)^c^	Oomen et al. (1992)
				22^b^	3 × >100 eggs^d^	OECD 75 (2007), Oomen et al. (1992)
*C. virginianus*	Microbial	Gelatin capsule	24.1–25.1 °C; 56–72 % RH; 8 h L/16 h D	14^b^	1 × 5♂, 1 × 5♀	OPPTS 850.2100 (1996b), FIFRA Subdiv E 71-1
*M. musculus*	Microbial	Gavage	19–25 °C; 30–70 % RH; 12 h L/12 h D	14^b^	1 × 5♂, 1 × 5♀	OECD 420 (2001)
*G. fasciatus*	5307 maize leaves	Direct consumption	17–19 °C; 16 h L/8 h D	5	4 × 10	OPPTS 850.1020 (1996a)
*I. punctatus*	5307 maize grain	Fish feed	23–27 °C; 16 h L/8 h D	28	4 × 20	ASTM (2004)

*RH* relative humidity; *L/D* light/dark period each day, *OPPTS* US EPA Office of Prevention, Pesticides and Toxic Substances [currently known as Office of Chemical Safety and Pollution Prevention (OCSPP)], *OECD* Organisation for Economic Co-operation and Development, *ASTM* American Society for Testing and Materials, *FIFRA* Federal Insecticide, Fungicide and Rodenticide Act (United States)

^a^Organisms per treatment: replicates × individuals per replicate

^b^Single or short-term dose: duration includes observation period after final dosing/exposure

^c, d^Independent studies

### Effects of eCry3.1Ab on surrogate non-target organisms

Tables 1 and 2 summarize the species and life-history stage tested, the functional groups represented, the routes of test organism exposure to eCry3.1Ab, the sample size, and the duration and physical conditions of the study. Species were chosen to comply with regulatory requirements in countries where cultivation approvals for Event 5307 maize may be sought. The species also fulfil many of the criteria for choosing laboratory test organisms to provide reliable information for ecological risk assessment (Romeis et al. 2013).

Each study followed a relevant guideline or protocol from organizations such as the Organization for Economic Co-operation and Development (OECD) and the United States Environmental Protection Agency (US EPA) (Table 2). These guidelines specify sample sizes, test endpoints and criteria to determine the validity of the study. All studies except for the bees met validity criteria for positive and negative control mortality. The study guideline for bees by Oomen et al. (1992) indicates that control mortality should not be, “generally above 15 %.” While the control mortality exceeded this minimum acceptable level, the studies were still informative for evaluating the effects of eCry3.1Ab on brood development because the test concentration that the bees were exposed to was much greater than the concentration produced in 5307 maize pollen. The data were more robust the closer the control mortality was to 15 %. In some studies, physical conditions sporadically varied outside the set limits; however, in no case was the study deemed to have breached the validity criteria of the protocol.

In each study, a negative control group was exposed to the same diet, vehicle, or test solution as the test group without the addition of eCry3.1Ab. In some instances a positive control treatment was included. In each case the reference substance used to produce the positive control treatment is expected to have effects on the organism being tested. The positive control treatment provides confirmation that the organism was exposed to the diet, vehicle, or test solution.

The composition of the diets used to expose test organisms are summarised here. Full details of the diets and the physical attributes of the test systems are available on request. The *Coleomegilla maculata* diet was a mixture of 50 % eggs of the moth *Ephestia kuehniella* and 50 % bee pollen collected from various flowers. The diet for the *Orius laevigatus*, *Aleochara bilineata* and *Poecilis cupreus* studies comprised a blend of roughly 46.5 % minced beef, 46.5 % lamb’s liver, 4.6 % yeast flakes and 2.4 % clear honey by weight. The beef and liver were cooked in an oven at 72 °C for 30 min and in an 800 W microwave oven on full power for 5 min to denature enzymes that might degrade the eCry3.1Ab. These components were liquidised and blended with a mixture comprising 62.3 % whole beaten egg, 9.3 % sucrose, 0.4 % Nipagin M preservative and 28 % purified water by weight. The finished diet comprised 57 % of the meat and liver mixture and 43 % of the egg and sugar mixture by weight.

Honeybees were exposed to eCry3.1Ab via a 50 % weight by volume sucrose solution. The test diet in the *Gammarus fasciatus* study was leaf discs of 5307 maize that had been soaked for 4 days in the same water as used in the test system. A control group was exposed to leaf discs from non-transgenic maize near-isogenic to 5307 maize. Bobwhite quail were exposed via gelatin capsules containing eCry3.1Ab, and mice were exposed to eCry3.1Ab via gavage using carboxymethylcellulose as the dosing vehicle.

Catfish were exposed to eCry3.1Ab via a standard catfish diet prepared from 41 % grain derived from 5307 maize. The control group was fed a similar diet prepared from grain derived from non-transgenic maize near isogenic to 5307 maize. Finally, earthworms were exposed via an artificial soil comprising 10 % sphagnum peat, 20 % kaolinite clay, 69.8 % quartz sand and 0.2 % calcium carbonate by weight.

Table 3 lists the concentrations of eCry3.1Ab to which the organisms were exposed in the effects tests and the endpoints measured. Several considerations were used to select the test concentrations, including regulatory requirements, the predicted exposures in the field, the desire to model worst-case exposures and apply conservative assumptions and the practical limitations of the test system. The nominal eCry3.1Ab test concentration is given when the test substance was supplied in a single dose by gelatin capsule, by gavage or in aqueous solution prepared fresh daily for the duration of exposure. When plant material was used for exposure, the percentage incorporation of plant material in the diet is indicated.

**Table 3:** Summary of results of effects tests of eCry3.1Ab

Test organism	Concentration or dose	Diet analysis	Endpoint	Positive control	Control (±SD)	Treatment (±SD)	Statistical test
*C. maculata*	353 μg eCry3.1Ab/g diet	Y (EWB)	Larval mortality	1	13.8 %^b^	2.5 %^b^	Fisher’s exact test
			Adult mortality		14.3 %^b^	10.5 %^b^	Fisher’s exact test
			Adult emergence		92.0 %^b^	97.4 %^b^	Fisher’s exact test
			Days to pupation*		12.0 (2.7)	9.6 (1.4)	ANOVA
			Days to adulthood		16.2 (2.5)	13.6 (1.4)	ANOVA
			Adult weight (g)		0.0118 (0.0023)	0.0142 (0.0027)	ANOVA
*O. laevigatus*	400 μg eCry3.1Ab/g diet	Y (EWB)	Mortality	2	0 %^b^	5 %^b^	Fisher’s exact test
*A. bilineata*	400 μg eCry3.1Ab/g diet	Y (EWB)	Fecundity^a^	2	838 (58)	824 (55)	*t* test
*P. cupreus*	400 μg eCry3.1Ab/g diet	Y (EWB)	Pre-imaginal mortality	2	10 %^b^	8 %^b^	Fisher’s exact test
			Mean adult weight (mg)^*^		65.7 (9.6)	52.7 (6.4)	t test
*E. fetida*	10.3 μg eCry3.1Ab/g soil	Y (EWB)	Mortality	None	0 %^c^	0 %^c^	Fisher’s exact test
			Change in weight		−9.1 % (2.3)	−7.2 % (5.3)	t test
*A. mellifera*	50 μg eCry3.1Ab/g sucrose solution	Nom (A/S)	Mortality (eggs)	3	25.5 % (11.7 %)	38.2 % (31.5 %)	ANOVA
			Mortality (larvae)		16.8 % (21.5 %)	2.3 % (1.7 %)	ANOVA
			Mortality (eggs)	4	20.03 % (13.73 %)	7.79 % (4.19 %)	Not analyzed
*C. virginianus*	900 mg eCry3.1Ab/kg bw	Nom (C)	Mortality	None	0 %^c^	0 %^c^	No test necessary
*M. musculus*	2,000 mg eCry3.1Ab/kg bw	Nom (G)	Mortality	None	0 %^c^	0 %^c^	No test necessary
*G. fasciatus*	100 % 5307 maize leaves	Nom (P)	Survival	None	80 % (14 %)	88 % (5 %)	t test
*I. punctatus*	41 % 5307 maize grain in feed	Nom (P)	Survival	None	100 %^c^	100 %^c^	No test necessary
			Mean weight gain (g)		115.91 (8.99)	111.21 (9.06)	t test
			Mean feed:gain ratio		4.50 (0.36)	4.79 (0.34)	t test

Nom (A/S): nominal concentration—test substance in aqueous solution consumed rapidly; Nom (P): nominal concentration—plant tissue; Nom (C): nominal concentration—test substance supplied in a gelatin capsule; Nom (G): nominal concentration—test substance supplied by gavage; Y (EWB): diet analyzed by ELISA, western blotting and bioassay with Colorado potato beetle

Positive control substances: 1 = 255 μg potassium arsenate monobasic/g diet; 2 = 0.01 mg teflubenzuron/g diet; 3 = 374.4 μg diflubenzuron/L diet; 4 = 75 μg fenoxycarb/g diet

* Statistically significant at 5 %

^a^Mean number of offspring per replicate of 10 males and 10 females

^b^Each replicate was comprised of a single individual. Therefore, the mortality endpoints were reported as either alive (0 % mortality) or dead (100 % mortality). Standard deviation was not reported

^c^Survival in these groups was 100 % therefore no standard deviation is reported

In studies where a microbially produced test substance was repeatedly supplied via an artificial diet, aliquots of treated diet were stored frozen and freshly thawed aliquots were supplied daily or every other day to the test species. Remaining aliquots were analyzed at the end of the in-life phase of the study to confirm the stability of eCry3.1Ab in the diet. The concentration of bioactive eCry3.1Ab in the diet was inferred from a weight of evidence based on the following assays: enzyme-linked immunosorbent assay (ELISA) to measure eCry3.1Ab concentration; western blotting to measure intactness of eCry3.1Ab; and a sensitive insect bioassay to confirm bioactivity. See Table 3 for details of diets that were analyzed.

### Concentrations of eCry3.1Ab in Event 5307 maize

Event 5307 maize was grown using standard local agronomic practices at three field locations in Illinois and one location in Minnesota. Concentrations of eCry3.1Ab in various tissues at several developmental stages were determined by ELISA and corrected for extraction efficiency (e.g., Nguyen and Jehle 2009). Tissues from nontransgenic, near-isogenic control plants were collected concurrently and analyzed in a similar manner to test for lack of eCry3.1Ab or interference from background substances.

### Estimated environmental concentrations of eCry3.1Ab from Event 5307 maize

Predicted exposures of non-target organisms to insecticidal proteins from cultivation of a transgenic crop are known as estimated environmental concentrations (EECs). For NTOs potentially exposed to eCry3.1Ab, EECs were estimated using data on the concentrations of eCry3.1Ab in relevant Event 5307 maize tissues. In order to calculate the EECs in a highly conservative manner, the highest mean eCry3.1Ab concentration reported for the relevant tissue at any single field trial location was used.

The diet of terrestrial invertebrates was assumed to be 100 % of the relevant Event 5307 maize tissue; thus, for these organisms, the EEC is equal to the highest mean concentration of eCry3.1Ab in that tissue measured at any single field trial location. These EECs are extremely conservative and present a worst-case exposure scenario because it is highly unlikely that the diet of these organisms is comprised solely of maize tissue (e.g., Raybould et al. 2007; Raybould and Vlachos 2011). Data on the environmental fate of eCry3.1Ab further confirm that the exposures tested were highly conservative. It has been demonstrated that eCry3.1Ab loses bioactivity in soil within 14 days; therefore, it is unlikely to persist or accumulate in the environment and result in longer-term exposures (Vlachos and Huber 2011).

For birds and wild mammals, exposure was estimated as a daily dietary dose (DDD), not as a concentration. DDDs were calculated from estimates of the food intake rates and body weights of seed-eating birds and rodents (Crocker et al. 2002) as described by Raybould et al. (2007). The worst-case (most highly conservative) DDDs for birds and mammals were calculated as 1.94 μg eCry3.1Ab/g body weight and 1.82 μg eCry3.1Ab/g body weight, respectively.

For farmed fish, the worst-case EEC was calculated assuming that fish feed is comprised of 30 % maize (NRC 1983) and that all the maize in the diet is Event 5307 grain. The worst-case exposure scenario also assumes that no eCry3.1Ab activity is lost during the preparation of the feed; this is unlikely as fish feed is heat-treated during preparation and eCry3.1Ab has been shown to lose biological activity after heat treatment (Vlachos and Huber 2011).

For aquatic invertebrates, potential exposure via pollen or plant debris transfer into waterways and runoff was considered. Dietary exposure to plant debris was considered to be the most conservative theoretical scenario, and therefore the worst-case EEC was taken as the highest mean concentration of eCry3.1Ab in leaves of Event 5307 maize.

### Risks to non-target organisms

A conservative formulation of the hypothesis that eCry3.1Ab in Event 5307 maize will not harm NTOs is that no NTO will be exposed to a concentration or dose of eCry3.1Ab greater than the highest concentration or dose of eCry3.1Ab that has no observed adverse effect (i.e., the no observed adverse effect concentration or level; NOAEC or NOAEL, respectively). The hypothesis of no adverse effects on NTOs from exposure to eCry3.1Ab in the field was tested by comparing the NOAEC obtained from the laboratory effect studies with the EEC estimates derived as described above. If the ratio EEC/NOAEC (the hazard quotient; HQ) is no greater than 1 for all groups of NTOs, the risks posed by exposure to eCry3.1Ab from cultivation of Event 5307 may be considered negligible (Raybould et al. 2007; Romeis et al. 2008; Raybould and Vlachos 2011).

## Results

### Estimated environmental concentrations of eCry3.1Ab

The fresh-weight concentrations of eCry3.1Ab in Event 5307 maize tissues are summarized in Table 4. eCry3.1Ab was detected in all tissues analyzed. For each tissue type, the highest mean eCry3.1Ab concentration at a single trial location at any developmental stage is reported. This concentration is used in the calculation of worst-case EECs.

**Table 4:** Highest mean fresh weight concentrations of eCry3.1Ab in 5307 plants grown at 3 locations in Illinois and 1 location in Minnesota

Tissue^a^	Highest mean at any site (μg eCry3.1Ab/g fresh wt.)	Growth stage
Leaf	51.74	Maturity
Kernel	5.53	Maturity
Root	6.48	Whorl
Pollen	0.22^b^	Anthesis
Whole plant	18.62	Whorl
Whole plant	4.89	Senescence

^a^For leaf, kernel, root, pollen and whole plant, N = 5 plants for each location

^b^Pollen samples collected from Illinois and Minnesota showed eCry3.1Ab concentrations either equal to or less than the LOQ (0.1 μg eCry3.1Ab/g dry weight pollen). Pollen collected from 5307 maize plants grown in Brazil showed higher levels of eCry3.1Ab in pollen than previously measured from the locations in Illinois and Minnesota. To be conservative the highest mean concentration measured in the Brazilian field trial (1 location) has been used here to calculate the EEC

Worst-case EECs of eCry3.1Ab for various NTO groups that could potentially be exposed via cultivation of Event 5307 maize are given in Table 5.

**Table 5:** Summary of estimated environmental concentrations (EECs) of eCry3.1Ab via 5307 maize

NTO group	Worst case exposure to eCry3.1Ab	
	Route	Concentration or dose
Foliar non-target arthropods	Diet 100 % 5307 maize leaves	51.74 μg eCry3.1Ab/g leaves
Soil-dwelling invertebrates	Diet 100 % 5307 maize roots	6.48 μg eCry3.1Ab/g roots
Pollinators	Diet 100 % 5307 maize pollen	0.22 μg eCry3.1Ab/g pollen
Wild birds	Diet 100 % 5307 maize kernels	5.53 μg eCry3.1Ab/g kernels 1.94 μg eCry3.1Ab/g body weight
Wild mammals	Diet 100 % 5307 maize kernels	5.53 μg eCry3.1Ab/g kernels 1.82 μg eCry3.1Ab/g body weight
Freshwater invertebrates	Diet 100 % 5307 maize	Concentration of eCry3.1Ab in Event 5307 maize leaf discs collected from greenhouse grown plants during the vegetative stage of development (not quantified)
Fish	100 % 5307 maize grain	30 % 5307 maize grain incorporated in fish feed

### eCry3.1Ab effects tests

Results of the effects tests with eCry3.1Ab are summarized in Table 3. In 8 of the 10 species tested, there were no statistically significant differences between the organisms exposed to eCry3.1Ab and those in the negative control group among any of the various endpoints assessed, including survival, weight, weight gain, fecundity, or development time. Among the *Coleomegilla maculata* exposed to microbially produced eCry3.1Ab there was no statistically significant difference in pupal mortality, adult mortality, adult emergence, number of days to adult emergence or mean adult beetle weight; however, there was a statistically significant difference in number of days to pupation. The individuals exposed to eCry3.1Ab reached pupation more quickly than the control group. This difference is not considered adverse and no statistical difference was reported in the subsequent measurement of the number of days to adult emergence. Among the *P. cupreus* larvae exposed to microbially produced eCry3.1Ab there was no statistically significant difference in mortality; however, there was a statistically significant difference in emergent beetle weight of the eCry3.1Ab-treated group when compared to the controls. On average the individuals exposed to eCry3.1Ab weighed 20 % less than the controls.

For the *P. cupreus* study, therefore, the NOAEC is less than 400 μg eCry3.1Ab/g diet based on the growth endpoint, or 400 μg eCry3.1Ab/g diet based on the mortality endpoint.

### Hazard quotients (HQ)

Table 6 lists the HQs for various functional groups. The HQ was calculated by dividing the worst-case EEC from Table 5 by the concentration or dose of eCry3.1Ab in the effects studies on species from the relevant functional group (Table 3); in all cases apart from the growth endpoint for *P. cupreus*, these values represent NOAECs or NOAELs as no effects were seen.

**Table 6:** Hazard quotients for organisms exposed to eCry3.1Ab via cultivation of 5307 maize

Test species	Functional group	EEC	NOAEC/NOAEL	Worst-case HQ
*C. maculata*	Foliar non-target arthropods	51.74 μg eCry3.1Ab/g diet	353 μg eCry3.1Ab/g diet	≤0.147
*O. laevigatus*	Foliar non-target arthropods	51.74 μg eCry3.1Ab/g diet	400 μg eCry3.1Ab/g diet	≤0.130
*A. bilineata*	Soil-dwelling invertebrates	6.48 μg eCry3.1Ab/g diet	400 μg eCry3.1Ab/g diet	≤0.016
*P. cupreus*	Soil-dwelling invertebrates	6.48 μg eCry3.1Ab/g diet	400 μg eCry3.1Ab/g diet <400 μg eCry3.1Ab/g diet	≤0.016 (mortality) >0.016 (development)
*E. fetida*	Soil-dwelling invertebrates	6.48 μg eCry3.1Ab/g diet	10.3 μg eCry3.1Ab/g diet	≤0.63
*A. mellifera*	Pollinators	0.22 μg eCry3.1Ab/g diet	50 μg eCry3.1Ab/g diet	≤0.004
*C. virginianus*	Wild birds	1.94 μg eCry3.1Ab/g bw	900 μg eCry3.1Ab/g bw	≤0.002
*M. musculus*	Wild mammals	1.82 μg eCry3.1Ab/g bw	2,000 μg eCry3.1Ab/g bw	≤0.0009
*G. fasciatus*	Freshwater invertebrates	100 % 5307 maize leaves	100 % 5307 maize leaves	≤1
*I. punctatus*	Fish (farmed and wild)	100 % 5307 maize grain	41 % 5307 maize grain	≤0.7

Using worst-case EECs, most of the HQs are well below 1. The exception is the *G. fasciatus* (freshwater invertebrate) which has a worst-case HQ of ≤1. *G. fasciatus* was provided a diet comprised of only Event 5307 maize leaf discs therefore the exposure represents at least 1× the worst case EEC. It is unlikely that *G. fasciatus* would consume a diet comprised solely of fresh Event 5307 maize leaves. The Event 5307 maize leaf discs to which the *G. fasciatus* were exposed were collected from Event 5307 plants at the vegetative stage of development. It has been observed that input of crop by-products into waterways is highest several months after field harvest (Jensen et al. 2010). Therefore, post-harvest plant debris will most likely be derived from senescent plant material. For all of the developmental stages from which Event 5307 plant material was analyzed, the mean concentration of eCry3.1Ab was the lowest in senescent tissues. Aging of Bt maize tissue is associated with rapid loss of activity of the insecticidal proteins as measured by sensitive insect bioassay (Jensen et al. 2010). Therefore, because *G. fasciatus* were exposed to a diet comprised solely of Event 5307 maize leaf discs collected from the vegetative stage of plant development this represents at least 1X the worst-case exposure scenario.

## Discussion

Apart from *P. cupreus*, no statistically significant adverse effects were observed following exposure to eCry3.1Ab, and worst-case HQs were well below 1 for most organisms. The general lack of adverse effects in studies in which representative indicator organisms were exposed to concentrations of eCry3.1Ab in excess of very conservative estimates of potential exposure corroborates the hypothesis that exposure to eCry3.1Ab via cultivation of Event 5307 maize will have little or no adverse effects on all NTOs, not only biological control organisms. Hence, the ecological risks from cultivating 5307 maize are negligible.

*Gammarus fasciatus* and *Ictalurus punctatus* tests involved exposure of the test group to plant material or a diet comprised of Event 5307 plant material. Studies where test groups are exposed to plant material can present a worst-case scenario by maximizing the percentage of Event 5307 plant material in the diet or collection of plant material from a particular plant developmental stage with high transgenic protein concentrations. To maximize exposure in the *G. fasciatus* study, the diet was comprised solely of Event 5307 leaf discs from the developmental stage with the reported highest level of eCry3.1Ab; in the field, aquatic invertebrates are likely to be exposed to aged Bt crop residues that have lost bioactivity (Jensen et al. 2010). For the *I. punctatus* study, exposure to Event 5307 maize grain was maximized by incorporating a high percentage of maize grain into the diet and all of the grain was derived from Event 5307 maize. The measured concentration of eCry3.1Ab in soil from the study with *Eisenia fetida* was less than nominal due to known difficulties with extracting Cry proteins from soil matrices (e.g. Palm et al. 1994).

For *P. cupreus*, HQs based on mortality were well below 1, even for the extremely conservative worst-case exposure scenario; however, on average *P. cupreus* exposed to eCry3.1Ab weighed less than the controls. Exposure to environmentally relevant concentrations of eCry3.1Ab is unlikely to result in risk to the ground beetle: the measured reduction in weight is slight, no effects on survival were observed, and the exposure scenario modelled in the study is extremely conservative. Furthermore, if the protection goal is preservation of predation of soil pests, small decreases in growth are unlikely to have adverse effects, particularly if more than one species provides that function (Raybould et al. 2011).

HQs are generally considered a conservative means to assess risk because the estimates of the NOAECs and EECs are not mitigated by environmental complexity. If HQs for a sufficiently broad range of surrogate species do not exceed a level of concern (here EEC/NOAEC = 1), negligible risk to NTOs from exposure to the insecticidal protein from cultivation of the particular crop may be concluded without further studies (Garcia-Alonso et al. 2006; Romeis et al. 2008). Reviews of field studies of Bt crops on NTOs indicate that conclusions of negligible risk to NTOs based on laboratory effects studies and conservative estimates of exposure are reliable (Romeis et al. 2006; Marvier et al. 2007; Duan et al. 2010); therefore, negligible risk to NTOs from exposure to eCry3.1Ab via cultivation of Event 5307 maize may be concluded with confidence from the laboratory effects data and conservative estimates of exposure based on concentrations of eCry3.1Ab in the crop.
